# Mental imagery for addressing mechanisms underlying motor impairments in children with attention deficit hyperactivity disorder (ADHD)

**DOI:** 10.3389/fneur.2024.1501871

**Published:** 2024-12-18

**Authors:** Arava Ron Baum, Eric Franklin, Yael Leitner, Amit Abraham

**Affiliations:** ^1^Department of Physical Therapy, School of Health Sciences, Ariel University, Ariel, Israel; ^2^Department of Physical Therapy, Sackler Faculty of Medicine, Tel-Aviv University, Tel-Aviv, Israel; ^3^The International Institute for the Franklin Method, Zurich, Switzerland; ^4^Dana-Dwek Children’s Hospital, Pediatric ADHD Clinic, Sourasky Medical Center, Ichilov, Tel Aviv, Israel

**Keywords:** ADHD, cognition, motor impairments, internal motor representation, timing, mental imagery, motor imagery

## Abstract

Children with attention deficit hyperactivity disorder (ADHD) exhibit various degrees of motor and cognitive impairments in fine and gross motor skills. These impairments impact social functioning, while also hindering academic achievement, self-esteem, and participation. Specifically, motor impairments are not fully addressed by current therapies. For example, approximately 50% of children with ADHD exhibit significant motor impairments, as per clinical measures, while the other 50% experience more impairments in motor planning, execution and control than do typically developed (TD) children. Such findings indicate that ADHD-specific mechanisms may be underpinning motor impairments. In this paper, we outline ADHD impairments in motor planning, execution, and control, and the potential role of two such mechanisms: internal motor representation and timing perception. Next, we suggest mental imagery as an approach for treating ADHD motor impairments, potentially through addressing internal motor representation and timing perception.

## 1 ADHD: definitions, etiology and diagnosis

Attention deficit hyperactivity disorder (ADHD) is the most common neurodevelopmental disorder among children and adolescents between the ages of 4–17 in the United States, with a prevalence of approximately 11% (1) and 3.4–10% worldwide (2–6). This clinical condition has a male–female ratio of 2.4–4:1 (1) and is characterized by persistent inattention and/or hyperactivity-impulsivity (1, 7). These symptoms impair academic, educational, social, and leisure activities (1–3, 7) thus impeding daily functioning, self-esteem, and well-being (4, 8).

The clinical presentation of ADHD involves additional motor and cognitive impairments, spanning motor planning, execution, and control (1, 9–11). Further, approximately 70% of children with ADHD present with a coexisting psychiatric (e.g., anxiety) or developmental disorders (e.g., developmental coordination disorder; DCD) (1, 12). Such co-occurrences may aggravate ADHD symptoms’ severity, lead to greater functional impairments, and make diagnosis and treatment more challenging (2, 6, 13–15). In this paper, we discuss ADHD impairments in motor execution, planning, and control, including the existing ambiguity in terms and definitions. We then highlight two mechanisms—internal motor representation of action and timing perception—as potentially underpinning ADHD motor impairments. Lastly, we suggest mental imagery as an appropriate approach for alleviating ADHD motor impairments through addressing those mechanisms in children and adolescents with ADHD.

## 2 Motor impairments in ADHD

**Figure 1:**
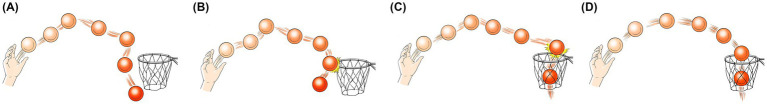
A metaphorical representation of ADHD population’s subgroups, based on clinical and motor control tests. Throw’s outcome represents clinical tests’ (e.g., M-ABC-2) scores: hit = ≥16th percentile, partial miss = 5th–15th percentile, complete miss = <5th percentile; Ball trajectory represents motor control (i.e., accuracy, response time) impairments: smooth = no impairments, jerky = impairments; **(A)** ADHD with significant motor impairments and motor control impairments (complete miss and jerky trajectory), **(B)** ADHD with probable/mild to moderate motor impairments with motor control impairments (partial miss and jerky trajectory), **(C)** ADHD without motor impairments and with motor control impairments (hit with jerky trajectory), **(D)** Typically developed (hit with smooth trajectory). Subgroups **(A,B)** are also labeled “ADHD with clinically detected motor impairmentsˮ and subgroup **(C)** is also labeled “ADHD with no clinically detected motor impairmentsˮ (Drawn by Eric Franklin).

Children with ADHD exhibit impairments in motor planning, execution, and control (11, 16, 17). Such impairments are manifested in both the gross (e.g., jumping, running, object manipulation) (18–23) and fine (e.g., manual dexterity) (22–26) motor skills. Further, impairments in motor planning and control include, among others, decreased accuracy and slower and variable movement time (20, 27, 28) (See Section 2.3). Impairments in motor execution are commonly diagnosed using standardized norm referenced motor tests in which the movements of the study group are compared to those of a normative sample, such as the Movement Assessment Battery for Children (M-ABC-2) (38) and the Bruininks-Oseretsky Test of Motor Proficiency (BOT-2) (30). These tests are, however, commonly used for developmental coordination disorder (DCD) diagnosis (7, 31). This could explain, at least in part, why children with ADHD are occasionally considered as having DCD co-occurrence, even without a comprehensive DCD diagnosis. Specifically, approximately 50% of children with ADHD are diagnosed—based on those standardized norm-referenced motor scales (8–10, 19, 23, 26)—with significant motor impairments, consistent with DCD (8–10, 19, 23, 26). However, current literature using this definition is inconsistent as per subgrouping those with “definite” versus “probable” scores (i.e., a score below 5th percentile or a score between 5th and 15th percentiles, respectively) (31). Further, some studies refer to children with ADHD as a homogenous group (22, 32–34), while others (26, 27, 35, 36) divide them into those with or without DCD/motor impairments based on these standardized motor tests. Interestingly, a few studies have shown that those children with ADHD but without scoring significant levels (i.e., ≥16th percentile) of motor impairments, compared to typically developed (TD) children, however, present decreased motor execution, planning and control (20, 27, 35, 37), thus demonstrating a specified motor profile (signature). These latter findings suggest additional underpinning mechanisms for ADHD motor impairments that are directly linked to ADHD etiology. Yet, the subgroup of children with ADHD as without significant motor impairments (as per standardized tests) is not adequately studied in ADHD research, despite exhibiting motor deficiencies. Therefore, it may be useful to refer to children with ADHD either with clinically detected (i.e., consistent with definite and probable DCD; <15th percentile) or without clinically detected (i.e., consistent without DCD; i.e., ≥16th percentile) motor impairments. Such a subgrouping not only distinguishes ADHD from its DCD co-occurrence, but also allows for better investigating ADHD-specific cognitive-motor mechanisms underpinning impairments in motor planning, execution and control. These current and suggested ADHD subgroupings are illustrated in Figure 1.

### 2.1 Motor execution impairments in ADHD

Motor execution impairments in children with ADHD are exhibited in a variety of tasks, such as walking, hopping, and ball and balance skills. Several studies found motor execution impairments in children with ADHD compared to TD, with only some of these studies subgrouping the ADHD population into with or without DCD (Table 1). These studies used standardized clinical tests (e.g., M-ABC-2, BOT2) (30, 38) and found that 30–60% of children with ADHD exhibited significant motor impairments in jumping, balance, manual dexterity, and ball skills (18, 22, 25, 32, 37) (Table 1). Further, studies that sub-grouped the ADHD population into with and without DCD showed that both subgroups exhibited motor impairments.

**Table 1:** Motor execution impairments in children with ADHD.

Participants	Subgrouping (based on DCD or levels of motor impairment)	Task/outcome measures	Main findings
ADHD (*N* = 24; *M* age: 8.88 ± 1.59 years, range: 7–12) and TD (*N* = 19; age matched) (32)	No	Single leg standing dynamic balance (Dynamic Y Balance Test)	ADHD group: decreased (*p =* 0.002) dynamic balance.
ADHD (*N* = 22; age range: 6–12 years) and TD (*N* = 22; age- and gender matched) (33)	No	Performance quality of 12 fundamental skills (TGMD2)	ADHD group: decreased (*p* < 0.05) performance in all 12 skills.
ADHD with DCD (*N* = 17; *M* age: 8.5 ± 1.25 years) and TD (*N* = 20; *M* age: 9 ± 0.95 years) (37)	Partial (not including ADHD without -DCD group); Based on: PANESS Test DCDQ	Kinematics of motor skills	ADHD+DCD group: greater impairments in inter-limb coordination [jumping jacks correct count (*p* < 0.001), jerk (*p* < 0.001)], balance [one-leg stance duration (*p* = 0.003), sway (*p* < 0.001)], timing [jumping jacks’ variability (*p* = 0.008)].
ADHD (*N* = 104 males; *M* age: 10 ± 1.4 years, range 7.8–12.11) and TD (*N* = 39) (25)	No	Motor Impairments (MABC, Purdue Pegboard)	ADHD group: lower total score (*p* < 0.025), ball skills (*p* < 0.005), and manual dexterity (*p* < 0.025); non-significant (*p* > 0.025) difference in balance scores.
ADHD with DCD (*N* = 13), ADHD without DCD (*N* = 9), and TD (*N* = 23) (total age range: 12–13 years) (26)	Yes M-ABC-2 (<5th percentile = with DCD)	M-ABC-2	Both ADHD subgroups: lower manual dexterity (*p* < 0.001), ball skills (*p* = 0.007), balance (*p* = 0.008) than TD; ADHD with DCD group: lower (*p* < 0.01) across all subsets compared to ADHD without DCD.

PANESS, Physical and Neurological Examination for Soft Signs; DCDQ, DCD Questionnaire; MABC, Movement Assessment Battery for Children; M-ABC-2, Movement Assessment Battery for Children-2; TGMD-2, Test of Gross Motor Development, 2nd Edition.

### 2.2 Motor planning and control impairments in ADHD

Motor planning and control impairments in ADHD include increased movement variability (20, 27, 28, 39, 40), timing deficiencies, timing variability and misperception (20, 27, 28, 34, 39), decreased movement accuracy (27, 28), increased execution time (27, 39, 40), and slow and variable reaction time (20, 28) (Table 2). Specifically, increased reaction time has been suggested to relate to impaired motor planning (11, 17) and to underlie altered inhibition intertwined with executive functions (11). Such impairments have been identified in a variety of tasks, including finger tapping (20, 27, 28, 40), jumping (39), and walking (34) (Table 2). Numerous studies have suggested that impairments in motor planning and control in children with ADHD could be attributed, at least in part, to impairments in timing perception (17, 20, 41), movement timing related circuitry in the cerebellum and basal ganglia (20), disturbed visuospatial working memory, inaccurate processing of the motor commands, dependence on visual feed-back during movement execution, and problems with internal representations of objects and visual space (20, 27, 35). Interestingly, a few studies that assessed motor planning and control in ADHD, and sub-grouped participants into ADHD with and without clinically detected motor impairments, found decreased motor control in both groups, compared to TD children (27, 35, 36). These findings support the role of ADHD itself—regardless of DCD—in motor planning and control impairments in children with ADHD.

**Table 2:** Motor planning and control impairments in children with ADHD.

Participants	Subgrouping (based on DCD or levels of motor impairment)	Task	Outcome measures	Main findings
ADHD (*N* = 16; age range: 6–19 years) and TD (*N* = 18; age range: 7–17 years) (34)	No	Walking with and without listening to text	Stride length and time variability	ADHD group: tendency (*p* = 0.09) toward high variability in stride length at baseline; Dual task improved (*p* < 0.004) stride time variability in ADHD group only.
ADHD (*N* = 28 males; *M* age: 10.2 ± 1.4 years) and TD (*N* = 23; *M* age: 10.8 ± 1.3 years) (40)	No	Finger tapping sequence; Go-No/Go task	Speed and time variability	ADHD group: slower (*p* = 0.07) movement, higher (*p* < 0.05) intra-subject time variability, slower/more variable (*p* < 0.005) reaction time in Go-No/go task. ADHD symptoms were predicted by finger sequencing speed (*p* < 0.05) and variability (*p* < 0.005), as well as reaction time variability (*p* < 0.005).
ADHD (*N* = 29; *M* age: 11.5 ± 0.5 years, age range: 8–15 years) and TD (*N* = 35; *M* age: 10.5 ± 0.4 years) (20)	No, although the whole sample was assessed for motor impairments (M-ABC-2)	Finger tapping Grip force of object Motor performance (M-ABC-2)	Movement variability and rhythmicity	ADHD Group: higher (*p* = 0.001) movement variability, impaired (*p* = 0.008) rhythmicity, and greater (*p* = 0.001) inter-tap interval coefficient of variation; lower (*p* = 0.0036) and variable (*p* = 0.003) grip force; Manual dexterity (*p* = 0.007) and aiming and catching (*p* = 0.042)
ADHD (*N* = 25; *M* age: 11.6 ± 1.1 years) and TD (*N* = 25; age and sex-matched). ADHD participants were grouped into: ADHD with motor impairment (*N* = 16) and ADHD without motor impairments (*N* = 9) (27)	Yes, based on MABC (<10th percentile: defined as ADHD with motor impairments)	Goal-directed arm movement with/without visual feedback	Path length ratio, peak acceleration, accuracy	Both ADHD subgroups: with visual feedback: higher path length ratio (*p* < 0.01) and peak acceleration (*p* < 0.01); without visual feedback: increased (*p* < 0.01) movement time, more (*p* < 0.01) errors, and higher path length ratio (*p* < 0.01) and peak acceleration (*p* < 0.01).
ADHD with DCD (*N* = 14), ADHD without DCD (*N* = 14), DCD only (*N* = 15), and TD (*N* = 15) (Sample’s age range: 8–12 years) (36)	MABC (<15th percentile: defined as ADHD with DCD)	Real and imaged visually guided point task	Speed-target width-difficulty index	ADHD and TD groups exhibited better (*p* < 0.005) MI ability compared to DCD group. Both ADHD groups: slower (*p* < 0.005) MI compared to TD group. The DCD only group: slower (*p* < 0.005) MI compared to all other groups.

M-ABC-2, Movement Assessment Battery for Children-2; MABC, Movement Assessment Battery for Children.

## 3 Suggested mechanisms underpinning motor impairments in ADHD

**Figure 2:**
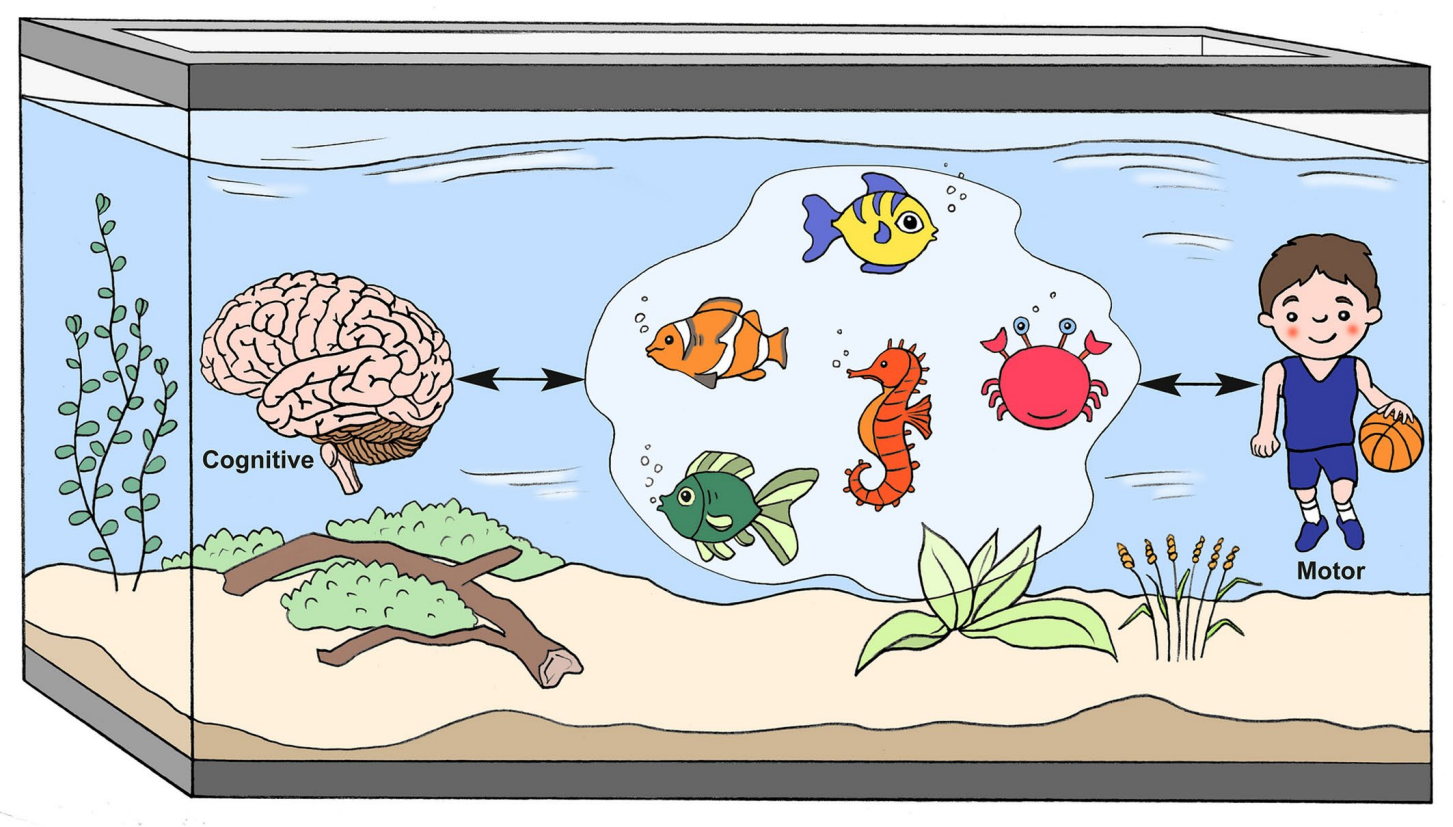
A metaphorical model of suggested cognitive-motor mechanisms underlying ADHD motor impairments. 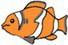, DCD co-occurrence; 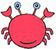, Executive Functions; 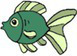, ADHD Core Symptoms; 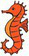, Timing; 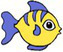, Mental Representations (Drawn by Eric Franklin).

The reasons for impairments in motor execution, planning, and control in children with ADHD are not fully understood, with numerous mechanisms being proposed (Figure 2). Among those mechanisms are DCD co-occurrence (9, 10, 21), ADHD core symptoms (9, 10, 22, 40), and executive functions (working memory, inhibition, and set shifting) (17, 42–44). Timing perception (20, 27, 39, 45) and internal motor representation of action (herein referred to as motor representation) mechanisms (20, 35, 41) have been less researched, although both potentially rely on—and thus share—the neuro-cognitive networks associated with motor planning (46–48). All of these mechanisms are likely to be intertwined and involve numerous brain regions (e.g., cerebellum, basal ganglia, thalamus, and frontal and prefrontal cortices) (49). For example, dopaminergic pathways involving dopamine and noradrenaline, particularly in the mesocortical, mesolimbic, and nigrostriatal areas, have been suggested to be associated with decreased attention, restlessness, impaired learning (50), executive functions (1, 43), motivational behavior (1), and timing (48, 51). A meta-analysis of functional magnetic resonance imaging (fMRI) studies of executive functions suggested that children with ADHD demonstrate cognitive-domain dissociated multisystem impairments in several right and left hemispheric dorsal, ventral, and medial fronto-cingulo-striato-thalamic and fronto-parieto-cerebellar networks, all of which mediate cognitive control, attention, timing, and working memory (48). Also, the inferior frontal cortex-parieto-cerebellar is one pathway that has been particularly associated with timing deficits (48).

Specifically, the high co-occurrence of DCD with ADHD in children suggests the role of the former in ADHD motor impairments, including learning and executing coordinated movements (31, 52, 53), thus resulting in low and slow motor performance (52–54). The role of ADHD core symptoms (inattention, hyperactivity, and impulsivity) in ADHD motor impairments is suggested by children with ADHD who are not clinically detected on the DCD standardized measures, and still exhibit motor impairments than TD children (26, 27, 35, 36). This clinical presentation is supported by research showing a positive correlation between ADHD core symptoms and impairments in motor execution and control (22, 40). A study comparing 42 children with ADHD and 42 age-matched TD (*M* age: 8.25 years) (22) found that level of attention and impulse control (based on Gordon Diagnostic System) predicted lower performance in balance and visual-motor control (Bruininks-Oseretsky Test of Motor Proficiency-BOTOMP) (55). Additional evidence for the possible interaction of ADHD core symptoms with ADHD motor impairments can be found in the positive effect of stimulant therapies on ADHD motor impairments (19, 34). Lastly, the role of executive functions in ADHD motor impairments is supported by literature reporting that children with ADHD exhibit deficits in brain regions (e.g., prefrontal cortex) that are involved in executive functions (4, 48, 56). Other studies have found positive correlations between deficits in executive functions and levels of ADHD motor impairments (17, 44).

Tracking the path of causality between either of these mechanisms—as well as the interaction among them—and ADHD motor impairments is, however, challenging for numerous reasons. For example, neuro-imaging findings do not perfectly correlate with motor impairments (49), ADHD core symptoms of inattention and hyperactivity do not always correlate with motor impairments (35), and medications for ADHD do not fully alleviate ADHD motor impairments (19, 34). The role of the other two mechanisms—motor representation and timing perception—is detailed below.

### 3.1 Internal motor representation of action

Internal motor representation of action (aka motor representation) refers to one’s mental depiction of an action, including its spatio-temporal characteristics and effect (57, 58). This cognitive image relies on, among others, one’s previous experience with, and understanding of, the task (57, 58). Motor representation is part of the internal forward model, a neural framework that simulates the body’s dynamic behavior and predicts its effect in relation to the environment (35, 57, 59, 60). Of note, using motor representation implies mentally imaging (simulating) the task in one’s mind, a process known as motor imagery. This simulation process underlies successful motor planning, execution, and control (57, 61–63). To date, the role of motor representation in motor impairments in children has been mostly studied with regards to DCD (36, 57, 64–66). However, deficits in motor representation could be also reflected in ADHD impairments in motor execution and control, such as timing misperception and reduced accuracy (20, 27, 41), as well as in cognitive tasks (35, 36). For example, children with ADHD (*N* = 32; *M* age: 9.9 years) exhibited significantly more errors than TD children (*N* = 31; M age: 10.2 years) on both visually interrupted and non-interrupted drawing tasks, with a greater difference in the former (41). However, no between-group differences were detected on two basic motor dexterity tasks. The authors concluded that ADHD motor impairments could be due to problems with motor representation more so than any problems with basic motor dexterity issues (41).

### 3.2 Timing perception

One likely mechanism associated with motor representation is timing perception (herein referred to as timing) of motor tasks (20, 28, 39). Children with ADHD exhibit altered brain activity in areas associated with timing (45, 48), which may underpin impaired rhythmicity (34, 37, 39), greater variability in movement time (20, 27, 28, 39), reduced accuracy (27, 28), and slower reaction time (17, 40). Further, children with ADHD exhibit greater difficulties in following cued (compared to non-cued) and changing (compared to constant) rhythmic movements (27, 37, 39). A study with 545 participants (age range: 5–19 years) evaluated timing properties of manual tasks (externally cued and non-cued maximal speed press-a-button, and tapping) in children with ADHD, their affected and non-affected siblings, and controls (28). Results showed that on the externally cued press-a-button task, children with ADHD and their affected siblings exhibited decreased accuracy and increased variability, while non-affected siblings showed increased variability only, compared to controls. On the non-cued maximal speed press-a-button task, both children with ADHD and their affected siblings exhibited slower and more variable performance, compared to controls. Non-affected siblings exhibited no differences from controls. On the self-generated tapping task, no between-groups differences were detected. The authors suggested that timing variability, accuracy, and speed could be all genetically linked to ADHD etiology, with the former being most clearly associated. Other studies investigated timing properties of jumping tasks in children with ADHD (37, 39). One study compared rope jumping under cued constant and variable tempo among ten children with ADHD (*M* age: 9.6 ± 1.27 years) and 10 TD (*M* age: 9.9 ± 1.54 years) (39). The ADHD group exhibited greater hand-foot deviation time (i.e., temporal synchronization) in both constant and variable tasks, as well as increased timing variability of the foot and rope whirling, separately. Another study investigated jumping jacks spatio-temporal kinematics in 17 children with ADHD with DCD (*M* age: 8.5 ± 1.25 years) and 20 TD children (*M* age: 9 ± 0.95 years) (37). Results showed that the ADHD with DCD group exhibited greater impairments in upper-lower limbs coordination (measured by number of correct jumps), higher jerk (i.e., reduced movement smoothness), and greater performance time variability, than did the TD group.

## 4 Current therapies for ADHD

Currently available pharmaceutical and non-pharmaceutical therapies for ADHD (67) focus on a multimodal approach (68, 69) that combines stimulants (e.g., Methylphenidate, Amphetamine) and behavioral therapy (e.g., parental and children training to support children’s positive behaviors (3), neurofeedback (70)). Such a combined approach is considered most effective (1, 4, 67, 71). As per pharmaceutical therapies, approximately 70–80% of individuals with ADHD have been found to respond positively (i.e., decreased symptoms and improved cognitive and behavioral functioning) to stimulants (1, 42, 67). Further, medications have also improved motor execution in children with ADHD, further supporting the role of ADHD core symptoms in motor impairments (19, 34). Studies into non-pharmaceutical—namely physical exercise—therapies for ADHD suggest some positive effects of multi-faceted exercise training programs on short- and long-term physical, cognitive, mental, and social well-being of children with ADHD (72, 73). Some studies, however, lack details regarding interventions’ characteristics (e.g., duration, exercise descriptors), and vary in methodologies (72–74). One study (75) assessed the effects of a moderate- to high-intensity physical activity program on fitness, motor skills, cognitive functions, and ADHD-related behavior in middle school age children with ADHD who did versus did not receive a 10-week physical activity program (including warm-up, progressive aerobic, muscular, and motor skills exercises and cool down). Results showed that the program improved locomotion and raw motor score (measured by the Test of Gross Motor Development, 2nd Edition; TGMD2) (76) and the number of push-ups). However, while current ADHD clinical guidelines refer to assessing co-morbidities, they do not address motor assessment or treatment (2, 4, 6, 15). Therefore, only half of children with ADHD with clinically detected motor impairments are eligible for physical therapy treatments designed to improve motor impairments (8).

Limited research only suggests cognitive training to enhance motor performance in children with ADHD (77, 78). One study (78) assessed the effect of a 12-week intervention of a problem-solving approach [Cognitive Orientation to daily Occupational Performance (Co-Op)] (79) on motor performance (measured by Bruininks–Oseretsky Test of Motor Proficiency; BOTOMP) (55) in children with ADHD (*N* = 6; age range: 7–12 years) (78). Results showed improvements in motor performance in 5 out of the 6 participants. One research-based approach that merges motor and cognitive components is mental imagery (80). Such an approach could serve as a promising avenue for treating cognitive-motor impairments in children with ADHD, as described below.

## 5 Mental imagery for addressing motor impairments in ADHD

One promising—yet understudied—approach for addressing motor (35, 81) and non-motor (82) impairments in children with ADHD is mental imagery. Mental imagery is the cognitive process of creating any (e.g., visual, kinesthetic, auditory) experience in the mind (83). Specifically, mentally imaging movement—known as motor imagery (MI)—is typically performed without overt physical execution (84–86). Using and relying on the motor task’s efference copy (i.e., internal model) (59), MI involves predicting and simulating somatosensory and functional consequences of the imaged movement or motor task (57, 58, 87). As such, MI can be used for practicing and enhancing motor planning, execution, and control (57, 88, 89), including when actual movement is impaired or not possible (due to injury, for example). The beneficial effect of MI on motor and non-motor spheres of performance have been widely documented (60, 85, 86, 90). Further, MI serves for studying motor representation as well as a method for updating and enhancing it (89, 91). Such updates following MI could possibly be due, in part, to the absence of motor and non-motor constraints (e.g., physical limitations, fatigue, pain, fear avoidance) that are often associated with physical execution (80). Recent literature has shown the benefits of mental imagery combined with actual movement (92–94). Such cognitive-motor dyads—namely dynamic motor imagery (83, 94, 95) or dynamic neuro-cognitive imagery (DNI) (93, 96)—have been shown to benefit motor performance in various populations. Their potential for ameliorating motor planning, execution, and control in children with ADHD through various mechanisms (e.g., attentional focus) is yet to be revealed.

The interaction between motor representation and timing is particularly relevant within the context of MI (57, 97). Given that both actual and imaged movements involve retrieval of task-specific spatio-temporal information from long term memory (61, 98), both types of movements rely on the same central mechanisms (88), including motor representation (99) and timing. As such, it is not surprising that actual and imaged movements exhibit spatial (i.e., brain activity) (100) and temporal (i.e., time duration; aka chronometry) (101, 102) similarities. Specifically, chronometry provides information about the individual’s temporal organization of the motor task and the ability to preserve it (101), which is one feature of motor representation (103). These spatial and temporal similarities are used for measuring and training cognitive-motor competencies in research and clinical settings.

Children with motor impairments, including ADHD, may experience difficulties in accurately mentally imaging motor tasks (65, 104). Such difficulties could be explained by, at least in part, deficient motor experience that results in compromised motor representation (58, 105). The majority of research into MI in children has focused, however, on DCD (65, 106) and found diminished MI ability in hand mental rotation and visually guided point tasks (57, 97, 104, 107). Such impairments in MI ability are suggested to support the internal deficit model in DCD (59, 97), in which there is a difficulty in “picturing” the desired movement and comparing it to the actual executed movement (106). Limited research into MI abilities in ADHD found reduced MI ability in children with ADHD with and without DCD, with the latter subgroup exhibiting better ability (35, 36). One study compared MI ability (measured by difficulty index, defined as the speed-target width-difficulty relation during real and imaged visually guided point task) among 4 groups of children (age range: 8–12 years): ADHD with DCD (*N* = 14), ADHD without DCD (*N* = 14), DCD only (*N* = 15), and TD (*N* = 15). Results showed that both ADHD groups and the TD group exhibited better MI ability compared to the DCD only group (36). Further interestingly, both ADHD groups exhibited slower MI compared to the TD group, however faster than the DCD only group. A follow-up study added measures of attention (The Test of Everyday Attention for Children; TEA-Ch (108)), Conners parents’ ratings (109), working memory (Cambridge Neuropsychological Test Automated Battery; CANTAB (110)) as well as a mental rotation task for the same four groups of children (age range: 7–12 years) (35). Results showed reduced accuracy in the mental rotation task in the DCD groups, compared to the TD group. On the visually guided pointed task, both ADHD groups, unlike the DCD only group, imaged movements conformed to the speed-target width difficulty relation. Interestingly, however, no correlations were detected between these results and either attention, sustained attention or working memory. The authors suggested that deficits in MI ability may underlie, or at least contribute to, some of the motor impairments in ADHD. While the association between MI abilities and ADHD motor impairments has not been empirically established, the results of these studies promote MI as a relevant therapeutic avenue for children with ADHD. Specifically, the similarity in MI abilities between children with ADHD with various degrees of motor impairments (i.e., with and without DCD) further supports MI’s suitability for the whole spectrum of ADHD motor impairments. This view aligns with previous recommendations for integrating MI within pediatric rehabilitation (111). Further, the impairments in MI ability detected in children with ADHD compared to TD children should not discourage clinicians from using MI in this population, given previous literature demonstrating gains in participants’ views towards MI and MI abilities, following MI training (112, 113).

As per MI training, a significant body of evidence supports the beneficial effects of MI training on motor execution and control (e.g., movement speed, accuracy) in children (60, 90), including those with DCD (114). One study (81) with 60 adolescents with ADHD (age range: 12–17 years) compared the effect of a single session of four types of interventions (MI, physical practice, combined MI and physical practice, and control) on a dart throwing task. No details regarding the MI contents were provided. Results showed no difference among groups in throwing scores at baseline. The retention (one-day) test showed that the combined intervention resulted in significantly better throwing scores compared to all other groups. The physical practice group had significantly better throwing scores compared to the MI and control groups, and the MI group had significantly better throwing scores than the control group. This study highlights that adolescents with ADHD may benefit from MI for improving motor execution. Another study (82) assessed the effect of a 6-month imagery-movement (“attention education”) training on attention control, using the Conners’ continuous performance test (CPT) for vigilance and attention (115) in 30 children (age range: 6–9 years) with ADHD (intervention: *N* = 17; control: *N* = 13). The intervention involved teachers’ participation in guiding the children in attentional control strategies, including 25 exercises of visual, kinesthetic, and auditory MI, and transferring them into academic activities. Some of the exercises included actual movement. Results showed that the intervention group improved in CPT reaction time compared to the control group. Of note, however, no studies to date have explored the simultaneous combination of imagery and movement for addressing motor and cognitive impairments in children with ADHD.

## 6 Conclusion

Children with ADHD exhibit various degrees of impairment in motor planning, execution, and control, compared to TD children. Assessment of these impairments relies mostly on standardized functional motor tests. Such a reality may overlook children with ADHD who present—based on these clinical tests—no or non-significant impairments, and yet exhibit motor-functional impairments that impede social functioning. Timing perception and internal representation are two suggested—yet under researched—mechanisms to underpin ADHD motor impairments in ADHD. Deficiencies in these mechanisms may impact children’s ability to mentally image movement. If so, children with and without clinically detected motor impairments stand to benefit from using various subtypes of mental imagery. Integrating mental imagery within ADHD management may enhance motor skill planning, execution, and control. Such gains could be due to mental imagery addressing, among others, timing perception and motor representation. The potential of mental imagery in identifying and treating cognitive and motor impairments in ADHD advocates continued research into novel applications for ADHD management and treatment protocols.
